# Development of the Indonesian Version of the Consumer Access, Appraisal, and Application of Services and Information for Dementia Instrument into Indonesian and Validation on a Sample of Older Adults

**DOI:** 10.1097/jnr.0000000000000723

**Published:** 2026-01-09

**Authors:** Widiyaningsih, Anung AHADI PRADANA, Herry SUSANTO, Huei-Ling CHIU

**Affiliations:** 1 International PhD Program of Gerontology and Long-term Care, College of Nursing, Taipei Medical University, Taipei, Taiwan; 2 College of Nursing, Universitas Karya Husada, Semarang, Indonesia; 3 College of Nursing, Universitas Islam Sultan Agung, Indonesia; 4 School of Gerontology and Long-Term Care, College of Nursing, Taipei Medical University, Taipei, Taiwan

**Keywords:** CAAASI-Dem-INA, cultural adaptation, dementia literacy, older adults, psychometric testing

## Abstract

**Background:**

The rising global incidence of dementia is an escalating public health issue. In 2021, the rate of dementia cases in Indonesia had already risen to 27.9%. Dementia literacy, which refers to the ability to acquire, assess, and apply knowledge about dementia, is crucial for increasing public awareness and improving dementia care. However, obstacles persist in advancing dementia literacy owing to difficulties in obtaining information and a lack of awareness regarding the importance of dementia knowledge. Addressing these challenges is essential to enhance dementia care at a societal level.

**Purpose:**

This study was implemented to translate and adapt the Consumer Access, Appraisal, and Application of Services and Information on Dementia (CAAASI-Dem) instrument into Indonesian (CAAASI-Dem-INA) and to evaluate its psychometric properties to ensure it is a valid and reliable tool for assessing dementia literacy in Indonesia.

**Methods:**

In this cross-sectional study, a two-stage translation procedure followed by psychometric testing was used. A sample of 319 older adults aged 60 years or older was recruited from Semarang, Central Java, Indonesia, using a convenience sampling method. Data were analyzed using descriptive statistics and confirmatory factor analysis (CFA) to assess the validity and reliability of the translated instrument.

**Results:**

The results of the psychometric evaluation indicate that CAAASI-Dem-INA offers satisfactory validity and reliability. Moreover, the good model fit obtained in the CFA confirms the construct validity, while the Cronbach alphas obtained demonstrate strong internal consistency (.934), and composite reliability (.744–.930), further supporting the reliability of this tool.

**Conclusions:**

The CAAASI-Dem-INA is a valid and reliable tool for measuring dementia literacy among older adults in Indonesia. Thus, it represents an important addition to existing dementia literacy assessment tools and may be used to gain a comprehensive understanding of dementia literacy levels in Indonesia. As a tool to help assess and promote improvements in dementia literacy, the CAAASI-Dem-INA can contribute to improving care and support for the growing number of people affected by dementia in Indonesia.

## Introduction

The increasing prevalence of dementia, tied closely to global aging trends, has placed significant burdens on health systems, particularly in low- and middle-income countries. Currently, 60% of the estimated 55 million people living with dementia worldwide reside in those regions, with over 10 million new cases diagnosed per year globally (World Health Organization, 2023). In Indonesia, 1.2 million individuals with dementia were recorded in 2016, with projections estimating an increase to 2 million by 2030 and 4 million by 2050 (Nugraha et al., 2022). In addition, the prevalence of dementia had reached 27.9% by 2021 (Farina et al., 2023). This rise in dementia cases, coupled with the growing accessibility of information regarding its treatment and management, underscores the importance of improving public knowledge and literacy about this syndrome.

Improving dementia-related knowledge and literacy aligns with the WHO’s global action plan on dementia (2017–2025), which focuses particularly on enhancing dementia awareness as well as improving publicly available dementia information (World Health Organization, 2017). Health literacy is a crucial area of public health that involves obtaining, comprehending, and applying health-related information to allow individuals to make well-informed decisions about their health (Chen et al., 2022). Inadequate health literacy in older adults correlates with adverse health outcomes, increased hospitalization rates, lower rates of engagement in disease prevention and early detection practices, less-effective communication with health care providers and utilization of health care services (Choi et al., 2018).

The results of some prior studies indicate that older adults have relatively low levels of dementia literacy (Leung et al., 2019; Werner, 2004; Zhang et al., 2017). In Indonesia, research into dementia literacy among adults has revealed dementia knowledge to be poor, with more than half of participants self-identifying as being unfamiliar with the terms dementia and Alzheimer's (Farina et al., 2024; Nugraha et al., 2022). This situation is partly attributable to lack of awareness about dementia and the limited access available to dementia information/ services, which hinders not only early identification and diagnosis but also ongoing care provision for people living with dementia (Hansra et al., 2023). Consequently, lower dementia awareness leads to inadequate care for those with dementia, spanning early identification to ongoing treatment. Moreover, lower awareness results in stigmatization and negative attitudes toward those with dementia and increases the burden on caregivers and family members (Patel et al., 2021; Smith et al., 2023).

Dementia literacy is a term that encompasses related scientific literacy (knowledge of dementia) and cultural literacy (beliefs about dementia) as well as the capacity to acquire, comprehend, and apply related information (Choi et al., 2018). The Dementia Knowledge Assessment Scale (DKAS; Annear et al., 2017) for evaluating dementia knowledge, the Dementia Attitude Scale (DAS; O’Connor & McFadden, 2010) for examining attitudes towards dementia, and the Consumer Access, Appraisal and Application of Services and Information for Dementia (CAAASI-Dem; Doherty et al., 2020; Nguyen et al., 2022) for measuring the ability to acquire, assess, and employ dementia-related services and information are several of the numerous validated instruments available for assessing dementia literacy. Access to health information and services is a key indicator of the Active Ageing Index, designed to assess the ability of older adults to live independently and securely. However, key challenges faced in accessing dementia information and services include difficulties in identifying relevant information and accurately interpreting this information and higher susceptibility to inaccurate information (Dixon et al., 2022). Also, awareness of the need for assessments related to knowledge about dementia care is generally low (Giebel et al., 2024). Moreover, the diverse and fragmented nature of Indonesia’s population, spread across numerous islands, introduces unique challenges to dementia care that are further influenced by various cultural, linguistic, and socioeconomic factors. Therefore, acquiring and applying dementia knowledge is crucial to enhancing dementia literacy across Indonesia’s diverse landscape.

The CAAASI-Dem was created to assess self-perceived confidence in one’s capacity to obtain, evaluate, and utilize dementia services and information. Furthermore, the efficacious use of resources such as CAAASI-Dem has a role in helping individuals with dementia preserve their ability to make independent decisions (Doherty et al., 2020; Nguyen et al., 2022). This tool, initially developed and applied to measure dementia literacy in individuals with an interest in dementia in Australia (Doherty et al., 2020), was subsequently refined and validated as the reliable 24-item, five-factor CAAASI-Dem in a later study. The five factors of this scale include: evaluation and engagement (EE), readiness (R), social supports (SS), specific dementia services (SDS), and practical aspects (PA). The CAAASI-Dem was shown to have good overall goodness of fit indices and subscale internal reliability, satisfactory convergent validity, and adequate discriminant validity (Nguyen et al., 2022).

Literacy is a concept encompassing three dimensions: scientific literacy (i.e., dementia knowledge), cultural literacy (dementia-related beliefs), and the capacity to acquire, comprehend, and apply related information. In Indonesia, existing instruments such as the Dementia Knowledge Assessment Scale Indonesian version (DKAS-INA) and Dementia Attitude Scale Indonesian version (DAS-INA) are available to assess knowledge and attitudes toward dementia (Mulyani et al., 2023). However, no tool is currently available to evaluate the capacity to obtain, comprehend, and utilize dementia information. Notably, the CAAASI-Dem is designed to assess the competencies required by consumers, including patients, families, and caregivers, to effectively identify and navigate dementia care services and information (Smith et al., 2023). Although the DKAS-INA and DAS-INA focus, respectively, on knowledge literacy and cultural literacy, neither was designed to evaluate the capacity to acquire, comprehend, and apply information. Therefore, in this study, the CAAASI-Dem was targeted as a more comprehensive dementia literacy tool for Indonesia.

The goals of this study were to first adapt the CAAASI-Dem for the Indonesian context (CAAASI-Dem-INA) and then to evaluate its psychometric properties on a sample of older Indonesian adults. The validated CAAASI-Dem-INA is expected to be instrumental in improving dementia care strategies in Indonesia by enabling health care providers to better understand and address the specific needs of their older adult clients. In addition, the aggregated results generated by this tool may be used to guide policymakers in designing targeted interventions and support systems to enhance dementia literacy. Therefore, validating and widely implementing the CAAASI-Dem-INA tool are critical to effectively tackling the increasingly prevalent issue of dementia in Indonesia.

## Methods

A cross-sectional study was designed to assess the validity and reliability of the CAAASI-Dem-INA. Approval to translate and adapt the CAAASI-Dem was obtained by the study authors from the original authors of the tool. The institutional review board approved the study (N202305087). To ensure participant rights and privacy in this study, several ethical safeguards were implemented. The researcher explained the study purpose and its voluntary nature as well as obtained informed consent from all of the participants. The participants were able to withdraw from the study at any time without consequences. Confidentiality was maintained by securely storing data and limiting access to authorized personnel only. This study was conducted in two stages: (1) translation and back-translation, and (2) psychometric property testing.

### Stage 1: Translation and Back-Translation

The CAAASI-Dem was translated into Bahasa Indonesia utilizing a forward- and back-translation method. Three bilingual professionals proficient in English and Bahasa Indonesia and knowledgeable about the health care requirements of older adults translated the original CAAASI-Dem version into Bahasa Indonesia. Following the researcher and translator agreement with the translated content, the initial version of the CAAASI-Dem-INA was confirmed. Next, three additional experts in linguistics conducted a bilingual back-translation. A comparison was conducted between the original CAAASI-Dem and the back-translated version to identify any differences/discrepancies. The final version of the CAAASI-Dem-INA was achieved through consensus among academics and a linguist on the most suitable translation, considering the cultural context of the instrument. They carefully rectified grammatical, linguistic, and semantic elements. Furthermore, the researchers confirmed that the translation process from English to Bahasa Indonesia did not lead to any loss or distortion of meaning in the translated scale items. To validate the instrument, the study was pretested and then evaluated by a sample representative of the target demographic (30 older adults). Their feedback was then used to clarify any confusing or culturally misaligned issues, thereby ensuring the instrument was both intelligible and contextually appropriate for the general Indonesian population.

### Stage 2: Psychometric Property Testing

#### Sample and Administration

This study was conducted in Semarang City, Central Java, Indonesia, from June 2023 to August 2023 on a sample of older adult residents aged 60 years and above. In Indonesia, older adults are defined as individuals aged 60 years or older (Sani et al., 2022). Accordingly, the inclusion criterion was older adults who were Indonesian citizens and able to communicate effectively in Bahasa Indonesia. Otherwise qualified individuals who were either unable to read or had a known psychiatric illness history or cognitive impairment diagnosis were excluded. Based on the recommendation that sample sizes should be 3–20 times the number of items on the scale and given that the item-to-factor ratio in this study was ∼5, the required sample size was determined to be >200 (Mundfrom et al., 2005). Three hundred nineteen qualified individuals were recruited and enrolled as participants. All of the participants submitted valid responses, with no missing data. Thus, data from all 319 participants were used in the analysis.

### Measurements

#### Sociodemographic and Information Related to Dementia

A supplementary survey was conducted to assess participant sociodemographic characteristics and dementia-related information. The sociodemographic data included demographic profiles, socioeconomic status, and educational background. The dementia-related information included prior education about dementia, personal experience with dementia, and primary sources of health-related information.

#### Consumer Access, Appraisal, and Application of Services and Information on Dementia

The CAAASI-Dem tool is essential to facilitating the ability of individuals to access, assess, and utilize dementia-related information and services (Doherty et al., 2020; Nguyen et al., 2022). The 24-item version of the CAAASI-Dem used in this study was created by Doherty et al. (2020) and comprises five factors and 26 items. It has demonstrated acceptable reliability, achieving an internal consistency for each factor exceeding .7. A subsequent validation study conducted by Nguyen et al. (2022) confirmed the structural validity of the 24-items assessed on a 5-point Likert Scale, with responses ranging from 1 (“*Not at all confident*”) to 5 (“*Extremely confident*”) for 15 items, and from 1 (“*Strongly disagree*”) to 5 (“*Strongly agree*”) for 9 items, yielding a total possible scale score between 24 and 120, with higher scores signifying greater levels of confidence. The CAAASI-Dem encompasses five factors: evaluation and engagement (EE), readiness (R), social supports (SS), specific dementia services (SDS), and practical aspects (PA). It has exhibited overall goodness of fit indices with root mean square residual/RMR (.048); parsimony normed fit index/PNFI (.864); goodness of fit index/GFI (.988); adjusted goodness of fit index/AGFI (.985); normed fit index/ NFI (.985), and relative fit index/ RFI (.983). Moreover, the CAAASI-Dem has demonstrated good subscale internal reliability scores of .854–.938, satisfactory convergence with factor loadings of .64–.91, average variance extracted (AVE) values exceeding .05, and adequate discriminant validities of the five factors with correlations between factors ranging from .318 - .685.

#### The Dementia Attitude Scale

The DAS was originally developed to measure attitudes toward dementia among college students and direct care workers. It contains 20 items under two factors (“social comfort” and “dementia knowledge”) scored using a seven-point Likert Scale ranging from 1 (*strongly disagree*) to 7 (*strongly agree*). The internal reliability (Cronbach’s alpha = .83–.85) and convergent validity (*r* = .44–.55) of the DAS have been shown to be acceptable (O’Connor & McFadden, 2010). There are six reverse-scored items (2, 6, 8, 9, 16, and 17), and the total score is calculated by adding up the points from each item for a total possible scale score of 20–140, with higher scores associated with a better attitude toward dementia. The DAS focuses on the attitudes of respondents toward the experience of dementia, whereas the CAAASI-Dem examines not only attitudes but also the cognitive and psychomotor aspects of the respondent’s responses to dementia. The DAS was translated into Bahasa Indonesia, and the content validity index using the item-content validity index (I-CVI) of nursing students was .98, with good reliability testing as indicated by a Cronbach’s alpha of .779 (Mulyani et al., 2023).

#### The Dementia Knowledge Assessment Scale

The DKAS comprises 25 items designed to assess knowledge of dementia in the four domains of causes and characteristics, communication and behavior, care considerations, and risks and health promotion (Annear et al., 2017). Both the DKAS and CAAASI-Dem focus on individuals’ cognitive knowledge regarding the experience of dementia. It has shown acceptable to good internal consistency with Cronbach’s alphas of .85 for the overall scale and .65–.76 for the subscales. The discriminant validity of the DKAS revealed significant differences among different groups of respondents (Annear et al., 2017). The DKAS has five response options using a Likert Scale, including “*false*,” “*probably false*,” “*probably true*,” “*true*,” and “*I don’t know*.” Points are assigned as follows: two points for a statement labeled “true” if it is accurate, and zero points for a statement labeled “false” if it is inaccurate. One point is awarded for selecting “probably true” for a true statement and “probably false” for a false statement. Conversely, zero points are given for responding “true” or “probably true” to a false statement, for responding “false” or “probably false” to a true statement, and for responding “I don’t know.” Each question has a score range of 0–2, and the total possible scale score ranges from 0 to 50 points, with higher scale scores indicating a greater level of dementia-related comprehension. The internal reliability of the DKAS-INA earned a Cronbach’s alpha of .713, and the I-CVI was 1.00, indicating good construct validity (Mulyani et al., 2023).

### Data Collection and Procedures

The entire survey was conducted through direct surveys administered by the researchers, with the identification of older adults facilitated by public health services in Indonesia. The participants were asked to complete four questionnaires (sociodemographic and information-related dementia, CAAASI-Dem-INA, DKAS-INA, and DAS-INA) in sequence. Health care providers in the public health services supported the study by checking the completion of the questionnaires at submission. The time required to complete the questionnaire was ∼20 minutes.

### Structural Validity

A CFA was undertaken on a data set of 319 people to determine if the factor structure could be repeated. The normality of the data was checked using skewness, kurtosis, and the Kolmogorov-Smirnov test, with the results showing the data to be abnormally distributed. The CFA was conducted using an unweighted least squares estimation method (Koğar & Koğar, 2015), with standardized regression weights used to measure both the factor loading of each item and interfactor correlations. Data for the CFA were evaluated to determine the fit of the model using three fit indices: absolute fit indices, including RMR, GFI, and AGFI; comparative or incremental fit indices, including NFI and RFI; and PNFI as the last parsimony fit index (Alavi et al., 2020). An RMR value close to zero was used to indicate a good fit. A perfect fit was indicated when .97 ≤ NFI ≤ 1, .95 ≤ GFI ≤ 1, and .95 ≤ AGFI ≤ 1, and an acceptable fit was indicated when .95 ≤ NFI ≤ .97, .90 ≤ GFI ≤ .95, and .90 ≤ AGFI ≤ .95 (Koğar & Koğar, 2015).

### Convergent and Discriminant Validities

Pearson’s *r* correlation values were determined to assess convergent validity (Aziato et al., 2015; Shah et al., 2016), with correlation coefficients of >.70 indicating a strong relationship with high levels of reliability and validity; coefficients of .40–.69 indicating a moderate relationship with high levels of reliability and validity; and coefficients of .20–.39 indicating a weak relationship with moderate levels of reliability and validity. Correlation coefficients <.20 were interpreted to indicate a poor relationship with poor reliability and validity (Aziato et al., 2015). In this study, DKAS-INA and DAS-INA were used to measure the convergent validity of CAAASI-Dem-INA, as these tools are the currently available dementia literacy tools in Indonesia, and as no other study has investigated a translated version of the CAAASI-Dem.

Discriminant validity argues that items associated with similar concepts should correlate more closely than those associated with dissimilar concepts. Discriminant validity was tested in this study using AVE analysis. Comparing AVE values with correlation coefficients provided insights into whether a construct’s items explained more variation than other constructs. According to Fornell and Larcker, the AVE for each construct should be at least .50, and maximum shared variances (MSVs) should be lower than the AVE (Zait & Bertea, 2011).

### Internal and Composite Reliabilities

The internal reliability assessment in this study used Cronbach’s alpha coefficient with a minimum value of .6 to indicate instrument reliability to prevent the correction or elimination of certain variables within its contents. Cronbach’s alpha values in the following ranges, .60–.69, .70–.79, .80–.89, and ≥.90 were considered as offering questionable, acceptable, good, excellent reliability, respectively (Mohd Arof et al., 2018).

Internal consistency was measured in this study using composite reliability, as Cronbach’s alpha assumes all items are equally reliable and have equal outer loadings on the construct. Composite reliability addresses this limitation by accounting for the varying outer loadings of the items within the construct. Composite reliability ranks objects based on individual reliability, with composite reliability values ranging from 0 to 1, and higher composite reliability corresponding to higher overall reliability. Composite reliability levels ranging from .60 to .70 are considered acceptable, while levels <.60 indicate inadequate internal consistent reliability (Haji-Othman & Yusuff, 2022).

### Data Analyses

Data were analyzed using IBM SPSS version 26 and AMOS version 25, both developed by IBM (IBM Corp., Armonk, NY, USA). IBM SPSS Statistics was employed for descriptive statistical analysis, while confirmatory factor analysis (CFA) was conducted using AMOS software within the SPSS program. Before analysis, data were inspected for missing values and normal distribution. Continuous data were represented using mean and *SD*, and percentages and counts were used to represent categorical data. The psychometric properties examined for all three questionnaires included the internal consistency using Cronbach’s alpha and the structural validity using a principal component analysis or a CFA and convergent validity.

## Results

### Sociodemographic Characteristics and Dementia Literacy

Data were collected from 319 participants. The sample had a mean age of 66.18 (*SD* = 6.05) years and included 140 (43.9%) males and 179 (56.1%) females. Most (87.5%) of the participants used Bahasa Indonesia as their first language for communication; 84% lived with their family, while the remainder lived alone or in a nursing home; 86.5% stayed in a private home; and 87.5% lived in an urban area. In terms of economic status, 47% earned less than the minimum wage. Half (48.3%) had a high school education, while 38.2% held a university degree, and 13.5% had been educated to the elementary school level. Only 11.6% had received dementia education, while 9.1% had life experience with a family member with dementia or knew others with dementia (17.6%). One-quarter (25.1%) had provided care for people with dementia, and 46.4% accessed health information from the internet (Table 1).

**Table 1:** Participant Sociodemographic Characteristics (*N* = 319)

Factor	*n* (%)
Age (year; mean and *SD*)	66.18 (6.05)
Sex	
Male	140 (43.9)
Female	179 (56.1)
Communication (Indonesia as one’s first language)	
Yes	279 (87.5)
No	40 (12.5)
Family structure (Do you live with your family?)	
Alone	25 (7.8)
Family	268 (84.0)
Nursing home	26 (8.2)
Socioeconomic (Where do you live?)	
Private home	276 (86.5)
Others (rent, official resident, nursing home)	43 (13.5)
Socioeconomic (The character of your living area?)	
Rural area	40 (12.5)
Urban area	279 (87.5)
Socioeconomic (What is your economic status?)	
Under minimum wage	150 (47.0)
Around minimum wage	106 (33.2)
Greater than minimum wage	63 (19.7)
Education (What was your highest educational attainment?)	
Elementary school	43 (13.5)
High school	154 (48.3)
University	122 (38.2)
Education (Do you have experience with dementia-related education or classes?)	
Yes	37 (11.6)
No or Unknown of dementia	282 (88.4)
Life experience with dementia (Was a family member diagnosed with dementia?)	
Yes	29 (9.1)
No or Unknown of dementia	290 (90.9)
Did you know another close associate with dementia?	
Yes	56 (17.6)
No or unknown	263 (77.4)
Do you have experience providing care for a person with dementia?	
Yes	80 (25.1)
No or unknown	239 (74.9)
Information source (Do you usually access health information from the internet?)	
Yes	148 (46.4)
No	171 (53.6)
Information source (Where do you get health information?)	
Family member	140 (43.9)
Health professional provider	99 (31.0)
Online	37 (11.6)
Books/newspapers	8 (2.5)
Television	35 (11.0)

### Structural Validity, Convergent, and Discriminant Validities

**Figure 1:**
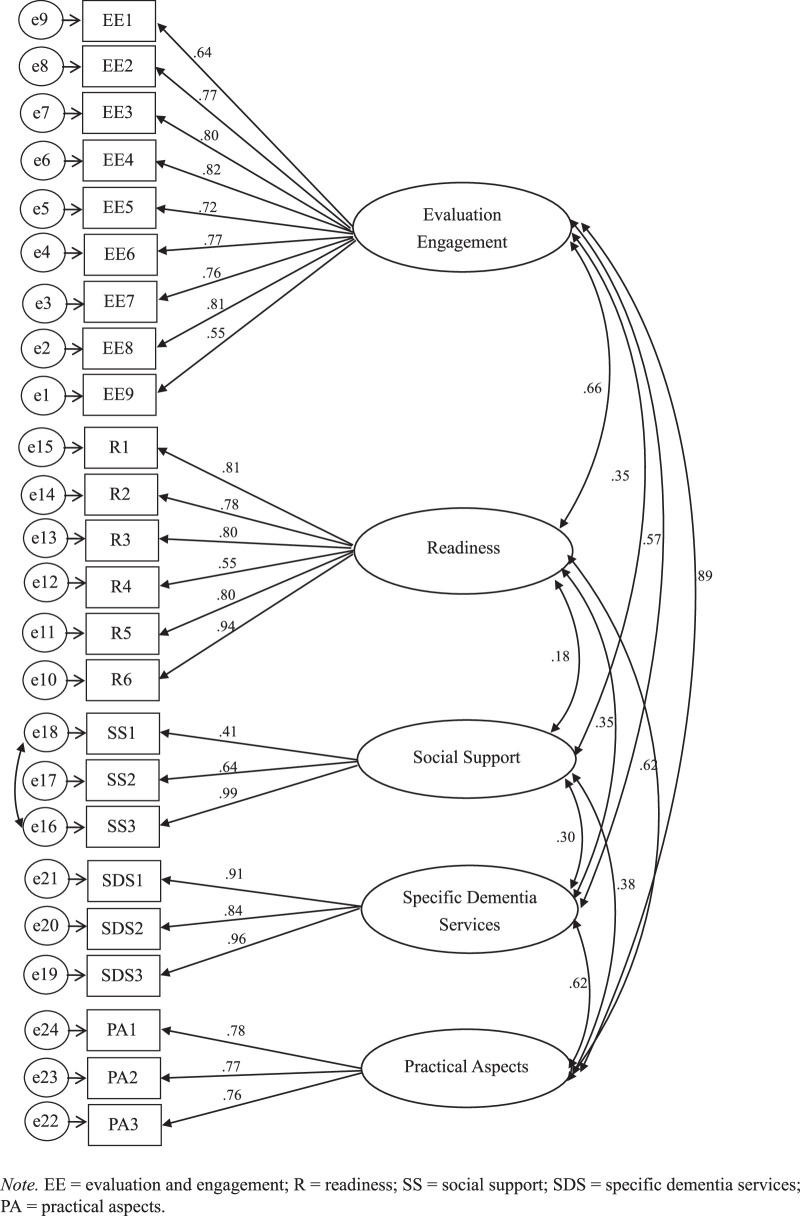
Best-Fitting Model of the Consumer Access, Appraisal, and Application of Services and Information on Dementia Instrument Into Indonesian and Its Parameter Estimates

Results of the CFA of the CAAASI-Dem-INA suggested a good model fit based on some indicators that exhibited only slight differences from the original instrument. We used the same method to examine the model fit with the original instrument. The 24 items and five factors based on a previous study (Nguyen et al., 2022), were examined using the CFA with a unweighted least squares estimation method. Factor EE had nine items, R had six items, SS had three items, SDS had three items, and PA had three items. Five items had a factor loading of <.70, with the lowest factor loading of .409, while one item (SS3) had a factor loading of >1, with a good model fit with an RMR of .077, GFI of .965, AGFI of .956, NFI of .955, RFI of .949, and PNFI of .838. To enhance performance on the measurement models with inadequate fit, a covariance within e16 and e18 was performed to improve model fit (Goretzko et al., 2024). The result was factor loading item SS3 changed to <1 (Table 2), with a slight change in the fit indices of PNFI to .834 (Figure 1). Results of the CFA showed small differences in fit indices between the model and the original CAAASI-Dem, with the fit indices RMR (.048); PNFI (.864); GFI (.988); AGFI (.985); NFI (.985); and RFI (.983). The AVE was .522 - .815 (> .50) below the AVE of the original CAAASI-Dem (.854–.938), which showed good discriminant validity and aligned with a previous study (Nguyen et al., 2022), but the maximum shared variances (MSVs) of two factors (EE and PA) were higher than the AVE (Table 3). Correlations between factors ranged from .179 to .903 compared with the .318–.685 range for the original CAAASI-Dem. The correlation among the factors of the CAAASI-Dem-INA indicates a low correlation between two factors (SS and R) and one high correlation factor (SDS; Table 4). The convergent validities of the two scales showed a moderate relationship with a high level of reliability and validity with the DKAS-INA (*r =* .423) and DAS-INA (*r* = .422) at *p* < .001.

**Table 2:** Standardized Factor Loadings for the CAAASI-Dem-INA

Item	Content	Factor				
		1	2	3	4	5
EE1	Deciding if dementia health information is relevant to me	.642				
EE2	Knowing whether to believe information about dementia	.771				
EE3	Understanding the health care advice that I am given about dementia	.796				
EE4	Questioning advice on dementia given to me by a health care provider	.820				
EE5	Comparing dementia health information from different sources	.723				
EE6	Discussing dementia with a health care provider by myself	.767				
EE7	Reading information from a health care provider by myself	.760				
EE8	Discussing very sensitive and personal issues about dementia with health care providers	.809				
EE9	Finding dementia health information by myself	.550				
R1	I have enough information about dementia to plan for future needs		.813			
R2	I have enough information about dementia to help me deal with current needs		.781			
R3	I have good quality information about dementia		.803			
R4	I can rely on at least one health care provider to help when there is an urgent need		.551			
R5	I have at least one health care provider who can give me advice about dementia-related health care needs		.801			
R6	Working out what dementia services may be needed in the future		.942			
SS1	If I need help I have people I can call			.409		
SS2	There are people I can spend time with			.636		
SS3	I have support from my community			.997		
SDS1	Accessing respite care				.905	
SDS2	Organizing an aged care assessment				.842	
SDS3	Organizing an advance care plan				.958	
PA1	Finding dementia-related health services					.780
PA2	Getting others to health care appointments					.765
PA3	Filling out forms on paper					.759

*Note.* EE = evaluation and engagement; PA = practical aspects; R= readiness; SDS = specific dementia services; SS = social supports.

**Table 3:** Internal Reliability and Discriminant Validity of the CAAASI-Dem-INA

Construct	Internal Reliability		Discriminant Validity	
	Cronbach's α	Composite Reliability	Average Variance Extracted	Cronbach's α
Evaluation engagement	.911	.917	.582	.787
Readiness	.898	.907	.625	.386
Social support	.777	.744	.522	.147
Specific dementia services	.928	.930	.815	.383
Practical aspects	.811	.812	.590	.787

**Table 4:** The Square Root of the AVE and Correlations Between Factors

Factor	SDS	EE	R	SS	PA
SDS	.903				
EE	.571	.763			
R	.353	.655	.790		
SS	.296	.353	.179	.741	
PA	.619	.887	.621	.373	.768

*Note*. AVE = average variance extracted; EE = evaluation and engagement; PA = practical aspects; R, readiness; SDS = specific dementia services; SS = social supports.

### Internal and Composite Reliabilities

The CAAASI-Dem-INA demonstrated strong internal consistency, with a Cronbach’s alpha coefficient of .934, indicating excellent reliability. The subscale scores ranged from .777 to .928, meeting the acceptable criterion of >.70 and aligning with the original version of the CAAASI-Dem (.854–.938; Doherty et al., 2020; Nguyen et al., 2022). The results suggest a relationship between the subscales of the 24 items in the CAAASI-Dem-INA. However, the correlations were not strong enough to show redundancy or duplication across the five domains. Composite reliabilities for the variables were all >.70 and indicated good reliability with a minimum value of .744 and a maximum value of .930 (Table 3).

## Discussion

The developed CAAASI-Dem-INA demonstrated satisfactory validity and internal consistency for use on older adults in Indonesia. It also showed a moderate and significant correlation with DKAS-INA and DAS-INA, indicating CAAASI-Dem-INA represents a comprehensive approach to acquiring, comprehending, and applying information related to dementia. The CAAASI-Dem-INA evaluates three essential elements, namely access to, appraisal of, and use of information and services (Doherty et al., 2020; Nguyen et al., 2022).

This validation of the CAAASI-Dem-INA for older adults in Indonesia demonstrated significant psychometric properties, evidenced by good fit indices from the CFA as well as robust convergent and discriminant validity. The test demonstrated high internal consistency and composite reliability, confirming this tool as both valid and reliable for measuring dementia literacy in the target population (older adults in Indonesia). This study is significant as it represents the first translation of a dementia literacy instrument designed to assess knowledge of dementia services and syndrome information among older adults in community, rather than educational course, settings. The rigorous translation process considered cultural and contextual factors to guarantee the instrument’s relevance in Indonesia. Most items demonstrated strong factor loadings with the exception of item SS1, which demonstrated a low factor loading. Nonetheless, item SS1 was preserved due to its significance in representing a unique aspect of the construct, hence maintaining the instrument’s content validity (Hayduk & Littvay, 2012). In addition, while some factors were identified as independent, the strong correlation for factor SDS suggests it represents a related construct (Cheung & Rensvold, 2002; Schmitt & Sass, 2011).

The factors of this instrument are designed to measure specific aspects of dementia literacy. Factor R assesses confidence in understanding and accessing dementia-related health care, factor SS evaluates access to community resources and services, and factor SDS indicates trust in services such as respite care, aged care assessment, and advance care planning (Nguyen et al., 2022). Items within factor SS represent community support, while those within factor R pertain to the dementia knowledge possessed by older adults. Factor SDS underscores the accessibility and reliability of dementia services, with findings suggesting that when older adults feel confident accessing these services, they are inclined to exhibit higher confidence in other factors as well. AVE values among the five factors confirmed strong discriminant validity, as each factor accounted for more variance in its indicators than it shared with other factors, which is consistent with prior research (Nguyen et al., 2022). However, the MSV values for two factors, EE and PA, exceeded their AVE values, suggesting potential discriminant validity concerns and indicating these two factors share a significant amount of variance, which may suggest conceptual overlap or redundancy between them. In other words, the items measuring EE and PA may not be sufficiently distinct, leading to challenges in clearly differentiating these constructs (Goretzko et al., 2024). The overlap between these constructs due to cultural or contextual factors in Indonesian population suggests further refinement of this instrument is necessary. Nevertheless, the moderate correlation found between EE and PA and their high reliability still demonstrated adequate convergent validity, particularly compared with the DKAS-INA and DAS-INA.

Factors such as community support dynamics and the role of caregivers in Indonesia may have influenced the findings. Indonesia is still in the process of developing its public long-term care system, which includes the program Indonesia Ramah Lansia dan Keluarga (Elderly and Family-Friendly Indonesia), which consists primarily of social security mechanisms and health care services. Puskesmas (Community health centers) and Posyandu (Integrated health posts) play significant roles in delivering long-term care, particularly within the health care sector, with the availability of related services varying significantly across provinces. The availability of day care, respite care, and institutional care remains limited in Indonesia, where caregiving for older adults, children, and other vulnerable community members is culturally and legally regarded as a family responsibility (Sani et al., 2022).

The strong correlation of the CAAASI-Dem-INA with both the DKAS-INA and DAS-INA further reinforces that this translated scale aligns with established measures, confirming its accuracy and dependability. The results indicate good internal validity across all subscales, which is consistent with the original version (Doherty et al., 2020; Nguyen et al., 2022) as well as other translated dementia instruments. The consistency observed across the 24 items of the CAAASI-Dem-INA supports the conclusion that all items measure the underlying construct of dementia literacy, specifically in relation to dementia services and information. Following a rigorous translation process, the CAAASI-Dem-INA was confirmed to be a valid and reliable dementia literacy assessment tool for assessing dementia services and information among the Indonesian older adult population. Both CAAASI-Dem-INA and the original CAAASI-Dem have demonstrated strong reliability and good overall model fit. However, some discrepancies were noted in the CAAASI-Dem-INA, including lower factor loadings for certain items, issues with discriminant validity (e.g., MSVs for EE and PA exceeding their AVEs), and a need for minor adjustments (covariance between e16 and e18) to further improve model fit. These differences may require further refinement to ensure robust measurement across cultural settings.

In this study, detailed insights are provided into the validity and high internal reliability of the proposed CAAASI-Dem-INA, positioning it as a dementia literacy tool for guiding targeted interventions and educational programs to address dementia literacy challenges within the community. This validated tool can be used together with other tools (e.g., DKAS-INA and DAS-INA) to guide evidence-based policies and practices and inform targeted public awareness campaigns in Indonesia by identifying gaps in the three dimensions of dementia literacy. It can also support the development of national dementia strategies by addressing cultural and contextual nuances (e.g. caregiver support and dementia-friendly programs); be used to monitor and evaluate the effectiveness of interventions related to dementia literacy; and be integrated into national health surveys to provide data for long-term planning.

### Study Limitation

This study was affected by several limitations. First, although the 24-item CAAASI-Dem-INA, consisting of five factors, demonstrated satisfactory reliability (internal and composite) and structural validity, some concern was raised with regard to its convergent and discriminant validities when tested on older adults in Indonesia. To address this issue, tests may be conducted on more diverse population samples or other established measurement instruments assessing similar constructs may be employed. Second, the significant variability in participant characteristics may account for the minor discrepancies found in the results. Participants in the original study were all enrolled in an online dementia course, while those in this study were older adults residing in the community with most lacking any previous dementia-related education experience. This distinction highlights the need to further validate the findings, especially with regard to convergent and discriminant validity concerns. In future studies, more diverse populations may be recruited to enhance the robustness of the findings.

### Conclusions

The CAAASI-Dem-INA developed in this study demonstrated satisfactory validity and internal consistency, while the CFA model of the CAAASI-Dem-INA showed good fit and dependability. However, it is important to resolve the potential discriminant validity problem, especially related to the elevated MSV values of specific components. Future related studies should focus on improving the measuring methodology and analyzing the correlations between subscales more thoroughly. The results of this study indicate the CAAASI-Dem-INA may be used to effectively enhance comprehension of dementia evaluations within Indonesia and offers significant perspectives for future study and therapeutic applications.
